# Prolonged elevated heart rate and 90-Day mortality in acute pancreatitis

**DOI:** 10.1038/s41598-024-59557-8

**Published:** 2024-04-28

**Authors:** Shan Xie, Fuxing Deng, Nuobei Zhang, Zhili Wen, Chenglong Ge

**Affiliations:** 1 Department of Gastroenterology, The Second Affiliated Hospital of Nanchang University, Nanchang, 330006 Jiangxi China; 2 Department of Oncology, Xiangya Hospital, Central South University, Changsha, 410008 Hunan China

**Keywords:** Heart rate, Mortality, Acute pancreatitis, Pancreatitis, Outcomes research

## Abstract

Prolonged elevated heart rate (peHR) is recognized as a risk factor for poor prognosis among critically ill patients. However, there is currently a lack of studies investigating the association between peHR and patients with acute pancreatitis. Multiparameter Intelligent Monitoring in Intensive Care IV (MIMIC-IV) database was used to identify patients with acute pancreatitis. PeHR was defined as a heart rate exceeding 100 beats per minute for at least 11 out of 12 consecutive hours. Cox regression analysis was used to assess the association between peHR and the 90-Day mortality. A total of 364 patients (48.9%) experienced a peHR episode. The 90-day mortality was 25%. PeHR is an independent risk factor for 90-day mortality (HR, 1.98; 95% CI 1.53–2.56; P < 0.001). KM survival curves exhibited a significant decrease in the survival rate at 90 days among patients who experienced a peHR episode (P < 0.001, 84.5% vs. 65.1%). We revealed a significant association of peHR with decreased survival in a large cohort of ICU patients with acute pancreatitis.

## Introduction

There are several reasons for the elevated heart rate in patients with acute pancreatitis, including anxiety, pain, medication effects, physiological regulation that increases cardiac output, excessive activation of the sympathetic nervous system, and hypoxia. Prolonged elevated heart rate is a risk factor for adverse cardiac events and poor prognosis in critically ill patients^1,2^. A multicenter retrospective study indicated that higher levels of heart rate are indicative of autonomic dysfunction and independently associated with poorer 1-year prognosis and lower survival rates in patients with cerebral hemorrhage^3^. Another study demonstrated that high heart rate increases the risk of cardiovascular mortality associated with elevated uric acid levels^4^. In non-hypotensive patients with acute symptomatic pulmonary embolism, elevated heart rate is associated with increased all-cause mortality and pulmonary embolism-related mortality^5^. There is a scarcity of guidance recommendations regarding heart rate management in acute pancreatitis, with limited evidence supporting these recommendations. Research has shown that continuous infusion of esmolol, a selective β-1 adrenergic blocker, can improve the outcomes of acute pancreatitis in rats and alleviate inflammatory response^6^. It can be inferred that sustained elevation of heart rate appears to be associated with the prognosis of acute pancreatitis. In patients with acute pancreatitis, tachycardia typically lasts for hours or days, but currently there is no available literature reporting the impact of the duration of tachycardia on the outcome of acute pancreatitis.

## Method

### Data source

This study utilized data from the Medical Information Mart for Intensive Care (MIMIC)-IV version 2.2, with permission obtained for the database (No. 0403000206). Informed consent was waived due to the de-identified nature of the data. MIMIC-IV is a publicly available dataset that contains comprehensive and de-identified information of patients admitted to the ICUs of Beth Israel Deaconess Medical Center in Boston, US, spanning the period from 2008 to 2019^7^. The MIMIC-IV dataset encompasses a total of 76,540 admissions.

Due to the utilization of only anonymized publicly available third-party data, this study was exempt from review by the human subjects committee. The reporting of this study adhered to the guidelines outlined in the REporting of studies Conducted using Observational Routinely-collected health Data (RECORD) Statement^8^.

### Patient selection

We included all adult patients with acute pancreatitis in the database who had their first ICU admission and a length of stay exceeding 24 h. Recorded heart rates were collected from the time of ICU admission, including the date and time of collection, and hourly medians were calculated. Any hours with missing heart rate measurements were identified, and patients were excluded if more than 20% of the hourly measurements were missing. To define prolonged elevated heart rate (peHR) episodes, we used the standard definition of 11 consecutive hourly heart rate measurements exceeding 100 beats per minute within any 12-h interval. In consideration of potential censoring due to death occurring during an episode of elevated heart rate, patients who had persistently high heart rates leading up to death were also classified as part of the peHR group using an extended definition. A sensitivity analysis was conducted excluding these patients from the peHR group, defining the episode based solely on the standard definition. Furthermore, a secondary analysis was performed by evaluating various combinations of duration and heart rate thresholds. This involved assessing different heart rate thresholds ranging from 90 to 120 beats per minute and durations ranging from 6 to 16 h.

### Data extraction

The information extraction from the MIMIC-IV database was performed using Navicat Premium software (version 16). Specifically, data were extracted from the following five categories: (1) Demographics, which included age, gender, race, and weight; (2) Vital signs, encompassing temperature, heart rate, blood pressure, and pulse oxygen saturation; (3) Comorbidities, consisting of conditions such as myocardial infarction, congestive heart failure, peripheral vascular disease, cerebrovascular disease, dementia, chronic pulmonary disease, rheumatic disease, peptic ulcer disease, liver disease, diabetes, renal disease, and malignant cancer; (4) Treatments, including the use of vasopressors/inotropes, beta-blockers, and mechanical ventilation; (5) Duration of hospitalization, follow-up survival status, and follow-up survival time. The database provided 1-year follow-up information for all discharged patients.

Laboratory variables were exclusively obtained from the initial 24 h following patient admission. In instances where multiple results were available, the average value was utilized. To mitigate potential bias, variables with missing values exceeding 10% were excluded. For variables with less than 10% missing values, the imputation process utilized the predictive mean matching method with distance aided selection of donors, which was implemented using the 'mice' package in the R software ^9^.

### Outcomes

We decided in advance to use the 90-day mortality as the primary outcome measure, considering it to be more meaningful than in-hospital mortality and less susceptible to being affected by underlying non-acute disease states.

### Statistical analysis

Statistical analysis included t-tests or analysis of variance (ANOVA) for continuous variables. Continuous variables were presented as mean ± standard deviation or median (interquartile range, IQR). For categorical variables, numbers (proportions) were used, and the analysis was conducted using chi-square tests, corrected chi-square tests, or Fisher's exact test. Kaplan–Meier survival analysis was employed to assess the incidence rate of major outcome events in distinct stratified groups based on prolonged elevated heart rate (peHR). Disparities were evaluated using log-rank tests. Cox regression analysis was performed to investigate the association between peHR and the 90-day mortality rate. Statistical analysis was performed using the software Stata 15.1 (https://www.stata.com/) and R 4.2.1 (https://www.r-project.org/) in the Windows operative system. Statistical significance was set at a p-value of less than 0.05.

### Ethics approval and consent to participate

The study was an analysis of a third-party anonymized publicly available database with pre-existing institutional review board (IRB) approval. The Institutional review boards at the Beth Israel Deaconess Medical Center (protocol 2001-P-001699/14) and Massachusetts Institute of Technology (protocol 0403000206) have approved the data collection and the use of MIMIC-IV for research purposes and granted waiver of informed consent. All methods were carried out in accordance with relevant guidelines and regulations.

## Results

### Patient characteristics

**Figure 1:**
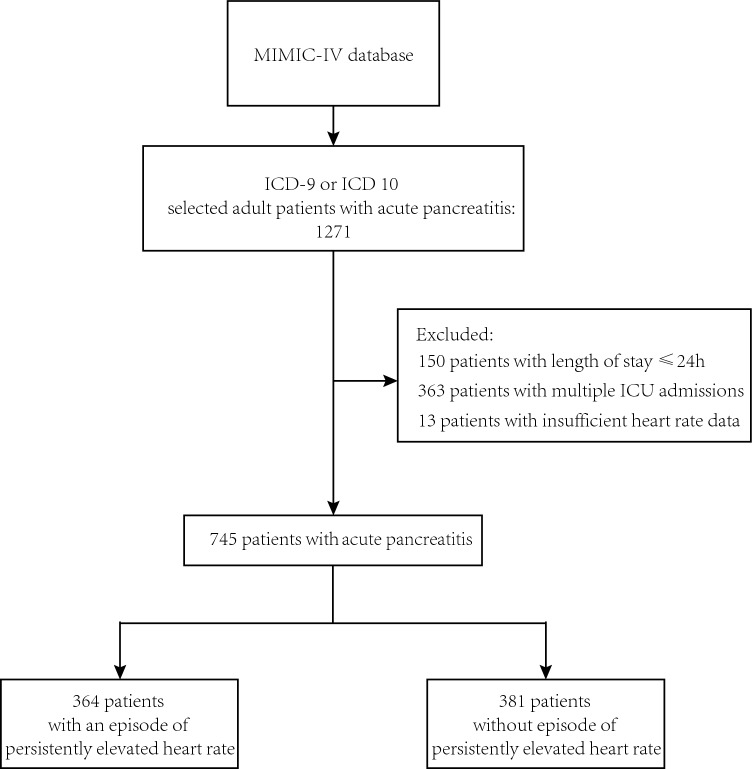
Patient inclusion flowchart.

**Figure 2:**
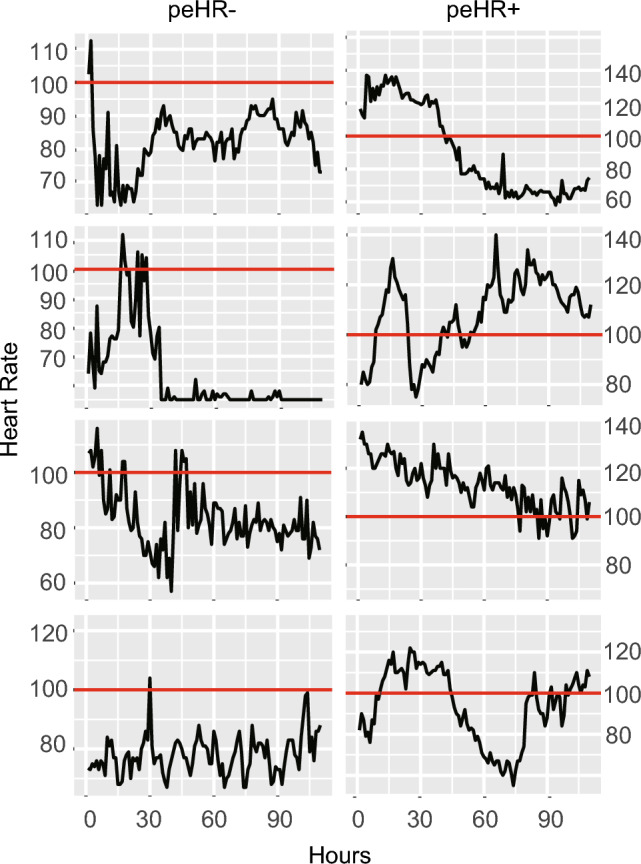
Example heart rate curves. The plots depict the hourly heart rate over time (x-axis). The left panel displays patients without prolonged elevated heart rate episodes (peHR), while the right panel shows patients with peHR (peHR +). In these examples, the red line represents the heart rate threshold of 100/min. A peHR episode was defined as having 11 out of 12 consecutive heart rates exceeding 100/min or above the red line in these examples.

Figure 1 depicts a flowchart illustrating the patient inclusion process. In total, 1271 patients were enrolled in the current study. Table 1 provides an overview of the patient characteristics. The average age was 58 years, with males representing 57% of the cohort. The predominant primary disease category was liver disease, observed in 28% of the cases. The mean Sequential Organ Failure Assessment (SOFA) score was 5. A total of 364 patients (48.9% of the study population) experienced a prolonged elevated heart rate (peHR) episode, with 312 patients meeting the standard definition and an additional 52 patients meeting the extended definition. The overall in-hospital mortality rate for the patients was 11%. Patients with peHR have a higher mortality rate compared to those with a normal heart rate (15% vs 8%, P = 0.005). Illustrative examples of heart rate tracings for patients with and without peHR episodes are presented in Fig. 2. Among all patients, heart rate-controlling medications were administered to 35% at any given time during their ICU admission. Specifically, 31% of patients in the group without peHR received such medications, whereas 39% of patients in the peHR group received them. Furthermore, 36% of patients received vasopressors or inotropes during their ICU stay, with 28% in the peHR- group and 45% in the peHR + group.

**Table 1:** Summary of patient characteristics.

Variables ^a^	Total (n = 745)	No peHR (n = 381)	peHR (n = 364)	P value
Age	58.38 (46.14, 72.95)	64.52 (51.58, 76.94)	52.54 (41.35, 66.74)	< 0.001
Sex, male	428 (57)	214 (56)	214 (59)	0.516
Weight	82.2 (70, 99)	80 (68.2, 97.5)	84.32 (71.18, 102.53)	0.006
SOFA score	5 (3, 9)	5 (3, 7)	6 (3, 10)	0.008
Heart rate	94.79 (81.11, 107.54)	83.48 (75, 92.12)	107.67 (99.99, 117.28)	< 0.001
MAP	80.38 (72.29, 91.21)	78.8 (71.31, 87.58)	82.39 (72.95, 93.46)	< 0.001
Temperature	36.96 (36.68, 37.39)	36.83 (36.61, 37.12)	37.17 (36.81, 37.61)	< 0.001
Spo_2_	96.29 (94.88, 97.81)	96.57 (95.15, 98.04)	95.92 (94.58, 97.61)	< 0.001
Co-morbidities				
Myocardial infarction	73 (10)	50 (13)	23 (6)	0.003
Congestive heart failure	142 (19)	88 (23)	54 (15)	0.005
Peripheral vascular disease	41 (6)	26 (7)	15 (4)	0.145
Cerebrovascular disease	44 (6)	22 (6)	22 (6)	1
Dementia	25 (3)	17 (4)	8 (2)	0.131
Chronic pulmonary disease	158 (21)	78 (20)	80 (22)	0.68
Rheumatic disease	25 (3)	14 (4)	11 (3)	0.771
Peptic ulcer disease	39 (5)	22 (6)	17 (5)	0.609
Liver disease	209 (28)	95 (25)	114 (31)	0.063
Diabetes	44 (6)	28 (7)	16 (4)	0.12
Renal disease	132 (18)	85 (22)	47 (13)	0.001
Malignant cancer	62 (8)	36 (9)	26 (7)	0.314
Ventilation use	381 (51)	155 (41)	226 (62)	< 0.001
Vasopressor/inotrope use	270 (36)	108 (28)	162 (45)	< 0.001
β-blockers use	261 (35)	118 (31)	143 (39)	< 0.001
Length of hospital stay	11.07 (6.47, 20.36)	7.87 (4.85, 14.62)	15.83 (8.81, 26.18)	< 0.001
Length of ICU stay	3.09 (1.81, 7.7)	2.09 (1.41, 3.59)	5.78 (2.78, 14.05)	< 0.001
In-hospital mortality	83 (11)	30 (8)	53 (15)	0.005
90-day mortality	186 (25)	59 (15)	127 (35)	< 0.001

*peHR*, prolonged elevated heart rate episode, *SOFA* Sequential Organ Failure Assessment, *MAP* mean arterial pressure.

^a^Numbers indicate median (IQR) or n (%).

### Outcome analysis

**Figure 3:**
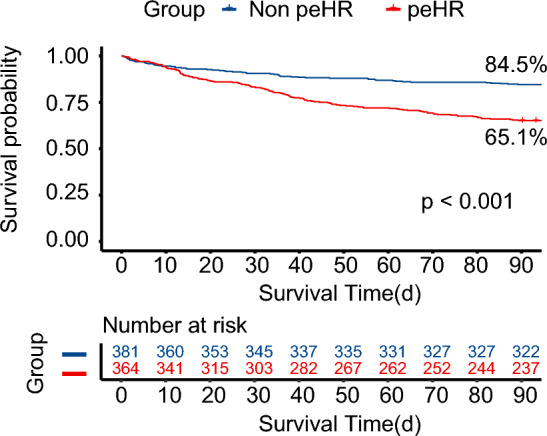
Kaplan–Meier plot. Survival curves were generated to compare patients with and without prolonged elevated heart rate episodes (peHR +) and (peHR −), respectively. The unadjusted survival curve showed a significant decrease in the peHR + group (dotted line, P < 0.001). Here, peHR represents prolonged elevated heart rate.

The overall 90-day mortality for the cohort was 25%. The Kaplan–Meier (KM) plot in Fig. 3 illustrates the unadjusted survival rates for patients with and without a peHR episode. The unadjusted survival rate was found to be significantly lower in the peHR + group (65.1% vs 84.5%, P < 0.001).

### The peHR is an independent risk factor for 90-day mortality

The results of the univariate Cox regression analysis revealed a significant association between the unadjusted peHR and all-cause mortality within 90 days of hospital admission (HR, 2.48; 95% CI 1.82–3.38; P < 0.001) (Table 2). Furthermore, in the multivariate Cox regression analyses, adjusting for potential confounding variables including age, SOFA score, MAP, temperature, presence of sepsis, and ventilation, a significant association between the peHR and 90-day mortality was observed (HR, 1.98; 95% CI 1.53–2.56; P < 0.001) (Table 2). The selection of these confounding variables was determined by their statistical significance level of P < 0.05 in the univariate analysis, along with consideration of our previous clinical experience.

**Table 2:** Univariate and multivariate COX regression analysis for death in patients within 90 days.

Characteristics	Univariate Cox regression			Multivariate Cox regression		
	Hazard ratio	CI 95	P value	Hazard ratio	CI 95	P value
peHR	2.48	1.82–3.38	< 0.001	1.98	1.53–2.56	< 0.001
Age	1.2	1.09–1.33	< 0.001	1	0.99–1.01	0.604
Sex	0.95	0.71–1.27	0.325			
Weight	1	0.99–1	0.419			
SOFA	1.13	1.09–1.17	< 0.001	1.05	1.02–1.08	0.001
Heart rate	1.01	1–1.02	0.011			
MAP	0.98	0.97–0.99	< 0.001	1	0.99–1.01	0.784
Temperature	0.67	0.54–0.85	0.001	1.15	0.93–1.42	0.203
Spo2	0.9	0.85–0.96	< 0.001			
Sepsis	2.48	1.72–3.59	< 0.001	1.53	1.16–2.02	0.003
Myocardial infarction	2.13	1.44–3.15	< 0.001			
Congestive heart failure	1.41	1.01–1.98	0.045			
Peripheral vascular disease	2.43	1.51–3.9	< 0.001			
Cerebrovascular disease	1.34	0.77–2.3	0.297			
Dementia	1.74	0.92–3.29	0.089			
Chronic pulmonary disease	0.97	0.68–1.38	0.869			
Rheumatic disease	1.41	0.69–2.86	0.345			
Peptic ulcer disease	1.52	0.87–2.68	0.143			
Liver disease	1.33	0.98–1.81	0.068			
Diabetes	1.01	0.55–1.85	0.984			
Renal disease	1.54	1.1–2.16	0.012			
Malignant cancer	2.27	1.52–3.39	< 0.001			
Ventilation	1.81	1.35–2.44	< 0.001	1.21	0.94–1.56	0.137
Length of hospital stay	1	0.99–1.11	0.988			
Length of ICU stay	1.01	1–1.03	0.005			

*peHR* prolonged elevated heart rate episode, *SOFA* Sequential Organ Failure Assessment, *MAP* mean arterial pressure.

### Propensity-score matching

**Figure 4:**
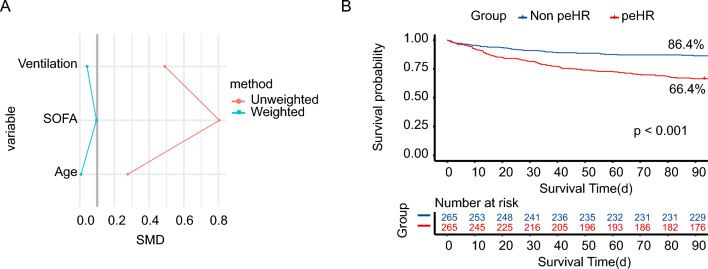
Kaplan–Meier plot after propensity score methods. (**A**) The plot displays the balance achieved in the crude and inverse probability weighted datasets, as indicated by the standardized mean differences (SMD) between the two groups (presence/absence of a prolonged elevated heart rate episode). Notably, the weighted dataset exhibits favorable balance with SMD values below 0.1. (**B**) The adjusted survival curve demonstrates a substantial decrease in the peHR + group (dotted line, P < 0.001). *peHR* prolonged elevated heart rate. *SMD* standardized mean differences.

To address the baseline differences, we performed propensity-score matching (PSM) with a caliper width of 0.2 logits of the standard difference. The patients were divided into two groups, peHR and non-peHR, using a 1:1 matching approach with the nearest neighbor. As shown in Fig. 4, the balance between the two groups was achieved in the weighted sample, with all covariates demonstrating standardized mean differences below 10%. Based on the statistical significance level of P < 0.05 in the univariate analysis and taking into account our previous clinical experience, the PS regression tree model identified age, ventilation, and SOFA score as the most influential variables. Following PSM, the KM survival curves analysis revealed that even after adjustment, the survival rate at 90 days remained significantly lower in the peHR + group (P < 0.001, 86.4% vs. 66.4%).

### Exploration of various thresholds

**Figure 5:**
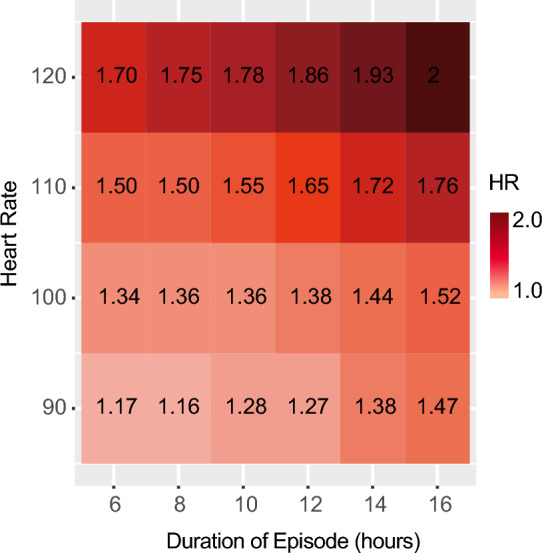
Hazard Ratio for various thresholds of heart rate and duration. We systematically explored multiple threshold combinations and calculated the HR for 90-day mortality using a minimally adjusted Cox regression model that considered age, ventilation, and SOFA score as covariates. The raster plot illustrates these HR values, with the number of patients meeting each set of criteria shown in parentheses. As heart rate and duration of prolonged elevated heart rate increase, the HR also increases, indicating a higher risk of mortality. *HR* hazard ratio.

In our analysis, we defined a peHR episode based on established clinical standards and prior published literature. As part of a post hoc analysis, we examined the impact of different heart rate thresholds and durations. Using a minimally adjusted Cox regression model (including age, ventilation, and SOFA score), we calculated the adjusted hazard ratio for mortality across various combinations of episode duration and heart rate thresholds. Figure 5 illustrates these results, with each square representing the corresponding hazard ratio for 90-day mortality both numerically and color-coded. The figure clearly demonstrates an elevated risk of death associated with higher heart rates and longer durations of peHR.

## Discussion

Our study aimed to investigate the association between peHR and survival outcomes in ICU patients with severe acute pancreatitis. To accomplish this, we conducted an analysis using a substantial sample size comprising patients admitted to the ICU. The dataset included comprehensive clinical information as well as 90-day survival data. Notably, both medical and surgical patients were included in our study to ensure representation across different specialties. It is important to highlight that the overall disease severity of the included patients was classified as severe, as indicated by a mean SOFA score of 5. Our study findings demonstrate a significant association between a 12-h episode of eHR > 100 beats/min and mortality within 90 days among ICU patients with acute pancreatitis. Importantly, this association persisted even after adjusting for several clinically relevant confounding factors, including age, ventilation, and SOFA score.

Furthermore, our analysis exploring various thresholds of duration and heart rate revealed that both parameters are independently related to survival outcomes (Fig. 5). These results highlight the potential importance of monitoring both the duration and heart rate in predicting patient prognosis in this population. These findings have implications for risk prediction and raise the question of a potential causal relationship. Although this observational study does not provide a definitive answer to this question, small-scale randomized trials have demonstrated a positive effect of medical heart rate control^6,10–12^. In our previous study, we discovered a significant association between the use of β-blockers and improved 28-day and 90-day mortality outcomes in patients with sepsis and septic shock^13^. However, there is limited literature available regarding the relationship between heart rate control and outcomes in acute pancreatitis. These results suggest that further investigation is warranted to explore the potential therapeutic benefits of heart rate management in improving outcomes for patients with acute pancreatitis. Previous evidence suggests that a subgroup of patients with tachycardia may also exhibit impaired microcirculation, and this combination of elevated heart rate and compromised microcirculation has been linked to unfavorable clinical outcomes^14^. One concern in critically ill patients receiving β-blockade is the potential for negative inotropic effects, which can impact cardiac function. In this context, Ivabradine presents an intriguing alternative for heart rate control as it offers the advantage of minimizing negative inotropic effects^15^. However, to provide clinically relevant recommendations, a more comprehensive dataset is required to assess the safety and efficacy of such interventions. Our study supports the exploration of interventions focused on safely reducing elevated heart rates, specifically in patients with acute pancreatitis who have persistently high rates exceeding 100 beats per minute.

Consistent with the findings of our study, a growing body of literature has demonstrated a clear association between elevated heart rate and increased mortality across a range of diseases. Zhang et al.^6^ conducted a study that revealed the potential of esmolol in attenuating lung injury and inflammation in rats with severe acute pancreatitis. Notably, compelling evidence suggests that effective control of a rapid heart rate can significantly improve the prognosis of patients afflicted with liver cirrhosis^16^, sepsis^12^, Marfan syndrome^17^, congestive heart failure^18^, and uncontrolled hypertension^19^. Given the potential clinical significance of heart rate management, our study contributes to the existing knowledge by establishing a research foundation for exploring the impact of heart rate control specifically in the context of acute pancreatitis. By shedding light on the potential benefits of heart rate regulation, our findings provide valuable insights into the potential therapeutic interventions that may positively influence patient outcomes in acute pancreatitis and warrant further investigation. These results emphasize the importance of considering heart rate as a modifiable factor in the management of acute pancreatitis and highlight the potential benefits of implementing strategies aimed at optimizing heart rate control in this patient population. Further research is needed to elucidate the underlying mechanisms and assess the impact of targeted heart rate interventions on improving outcomes in acute pancreatitis.

Heart rate, as a fundamental cardiovascular parameter, is profoundly influenced by medical interventions, and the modification of heart rate in ICU patients is a common practice in clinical settings. In our study, approximately 39% of patients with peHR received β-blockers, highlighting the frequency of medical heart rate modification in this population. However, despite the widespread use of these interventions, there is a paucity of data regarding optimal heart rate targets and their impact on patient outcomes. To address this knowledge gap, we aimed to contribute to the existing evidence base by utilizing data from the observational MIMIC-IV dataset. Our study sought to provide valuable insights into the relationship between heart rate management and clinical outcomes in acute pancreatitis. Nonetheless, it is important to recognize that observational studies have inherent limitations and cannot establish causality or provide actionable clinical guidelines. Moving forward, randomized controlled trials will be essential to further elucidate the efficacy and safety of different heart rate goals in ICU patients with acute pancreatitis. Such trials would not only help refine our understanding of optimal heart rate management strategies but also provide robust evidence to guide clinical decision-making and the development of evidence-based guidelines. By combining the insights gained from observational studies with the rigor of randomized trials, we can strive towards actionable and evidence-driven recommendations for heart rate management in acute pancreatitis.

However, our study has several limitations. Firstly, the retrospective design of the study imposes constraints on the findings, potentially impacting the strength of the conclusions made. Secondly, the utilization of a singular database in the study may restrict the generalizability of the findings to diverse populations or settings. Subsequent research endeavors could focus on corroborating these outcomes in alternate cohorts. Thirdly, due to the limited sample size in our study on acute pancreatitis and the need for propensity score matching analysis, we were unable to stratify acute pancreatitis based on etiological factors and severity.

## Conclusion

Our study findings reveal a significant correlation between a 12-h episode of elevated heart rate (eHR) exceeding 100 beats per minute and mortality within 90 days among patients with acute pancreatitis in ICU. These results emphasize the importance of monitoring both the duration and heart rate as valuable prognostic indicators in acute pancreatitis.

## Data Availability

All data and material were available at https://mimic.mit.edu/.
